# Supplementary figures and images for: Enhanced cytotoxic activity of natural killer cells from increased calcium influx induced by electrical stimulation
Source: PLoS One. 2024 Apr 18;19(4):e0302406. doi: 10.1371/journal.pone.0302406 (PMC11025832; doi:10.1371/journal.pone.0302406)

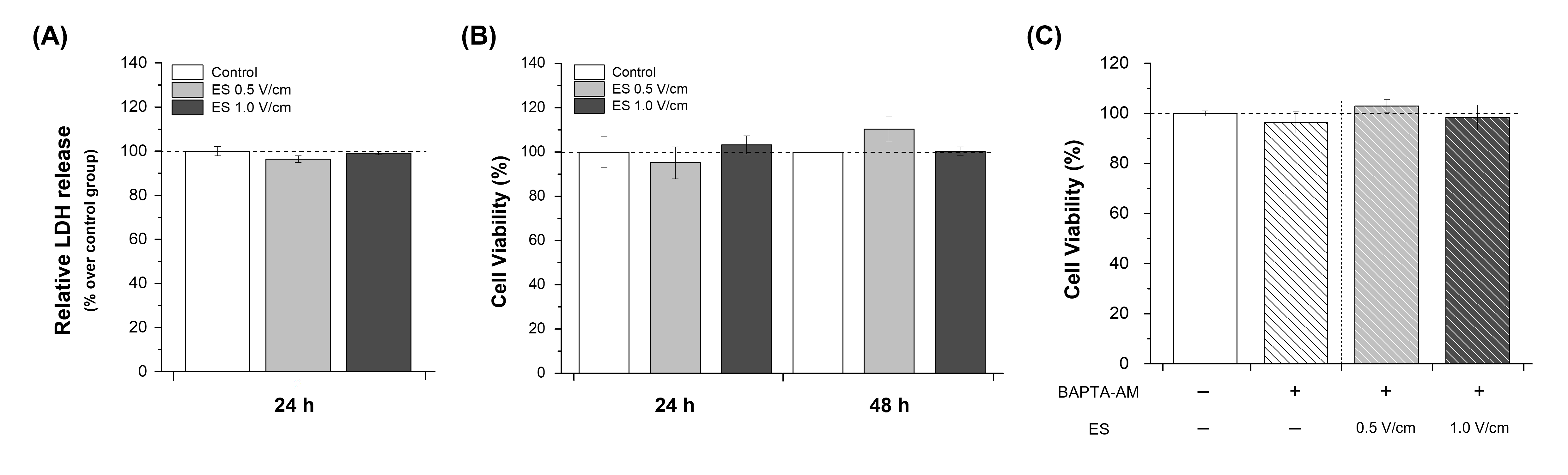

Supplement: S1 Fig — (A) LDH cytotoxicity assay after 24 h of incubation following electrical stimulation (1 h) (n = 3). Relative LDH release levels were calculated relative to the control group in terms of fold change. CCK-8 assays were used to detect (B) the long-term effects of electrical stimulation on cell viability after 24 h or 48 h and (C) the effects of BATPA-AM or electrical stimulation with BAPTA-AM on the viability of NK cells (n = 3). (TIF) [file pone.0302406.s001.tif]

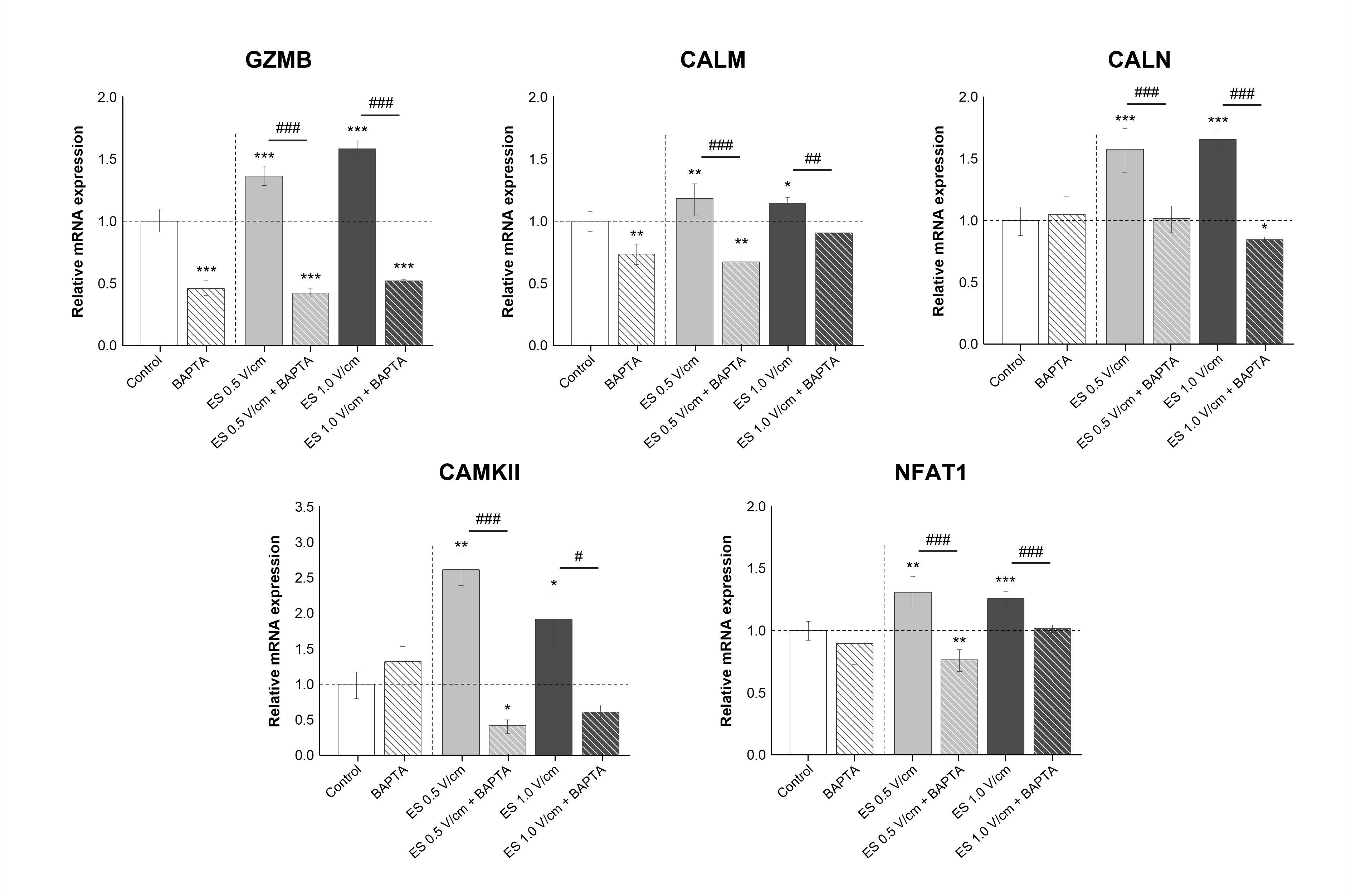

Supplement: S2 Fig — Relative gene expressions were normalized to GAPDH and expressed as fold change over the control group. One-way ANOVA and Tukey’s post-hoc test (R Studio program, http://www.rstudio.com/) were conducted for multiple comparisons (n = 3, * or #, P < 0.05; ** or ##, P < 0.01 and *** or ###, P < 0.001). (TIF) [file pone.0302406.s002.tif]

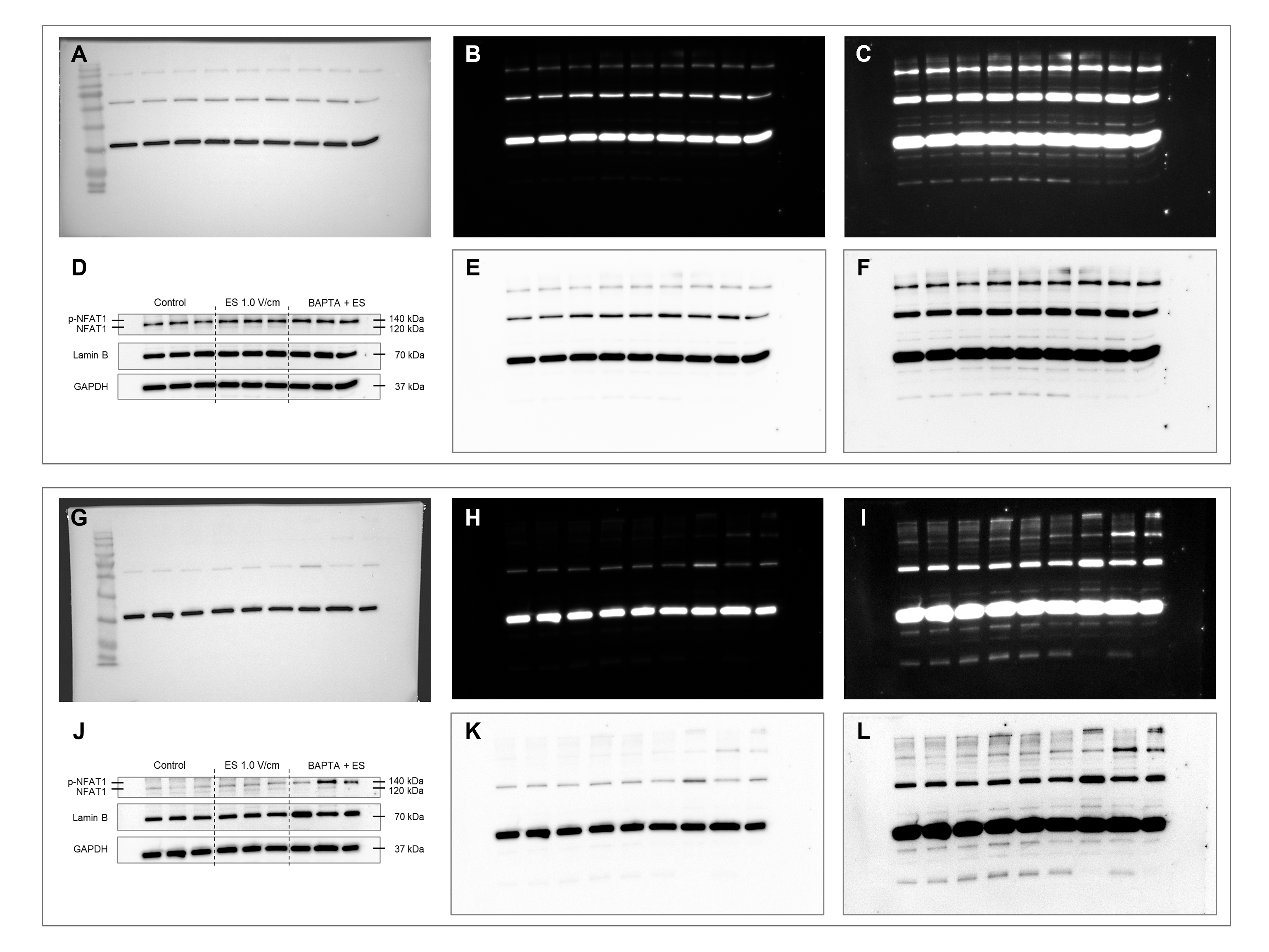

Supplement: S3 Fig — Full-length images of NFAT1, Lamin B as a nuclear loading control, and GAPDH as a loading control. Whole-cell protein sample prepared by RIPA lysis buffer (A)–(F), and nuclear extraction kit (ab113474, Abcam) (G)–(L); (A), (G) western blotting full images with marker; and (B), (C) and (H), (I) inverted, overexposed version of each full image; (E), (K) full-length image; (F), (L) overexposed version of full-length images; (D), (J) cropped version similar to that of Fig 5B presented in the manuscript. (TIF) [file pone.0302406.s003.tif]
