# Supplementary material for: Enhanced cytotoxic activity of natural killer cells from increased calcium influx induced by electrical stimulation
Source: PLoS One. 2024 Apr 18;19(4):e0302406. doi: 10.1371/journal.pone.0302406 (PMC11025832; doi:10.1371/journal.pone.0302406)
Supplement: Attachment — Submitted filename: Response to Reviewers Comments_PLOS ONE.docx [file pone.0302406.s004.docx]

November 27, 2023

We thank the reviewers for the insightful and constructive suggestions. We are addressing them below. The revised manuscript has been much improved. The reviewers’ comments are in *italic*, while responses are in plain text.

***Reviewer #1’s comments:***

*1) Can you provide more information on the specific electrical stimulation system used in the study? How was it designed and implemented to ensure accurate and controlled electrical stimulation of the NK cells?*

**Response:** As described in Fig 1A of Materials and Methods section, the electrical stimulation system consisted of two platinum electrodes (cathodes and anodes, 20 mm apart in parallel) on a lid above each well and a function generator [1–4]. The condition of stimulation was controlled by the function generator, and its performance was verified using an oscilloscope. It was designed to affect cells directly through the culture medium by inserting electrodes, which were fully applied throughout the well.

We revised the corresponding sentences at the line 86~92 in Page 5.

“ ~ The electrical system chamber was printed using a 3D printer (3DP-110F; Cubicon, Seongnam, Korea) fitted with a lid for standard 6-well plates (Fig 1A). The electrical stimulation system consisted of two platinum (Pt) electrodes (cathodes and anodes) (99.9% Pt wire with 0.3 mm diameter) at a distance of 20 mm in parallel (Fig 1). The condition of stimulation was controlled by the function generator. It was designed to affect cells directly through the culture medium by inserting electrodes, which were fully applied throughout the well. ~”

*2) How was the correlation between elevated intracellular calcium levels and NK cell activation determined? Were any other factors considered or controlled for in this analysis?*

**Response:** Previous studies have reported that applied electrical stimulation could induce changes in cell differentiation, proliferation, and migration, and the effects were mediated by the calcium ion [1–4]. In this study, the stimulation could induce an increased level of gene and protein expression of granzyme B (RT-qPCR, ELISA data), with an increase in intracellular calcium level (Fig. 5A) and an upregulation in calcium signaling-related proteins (Fig. 5B). As we conducted the experiments with the calcium ion chelator BAPTA-AM, the correlation between elevated intracellular calcium level and NK cells’ degranulation seemed to be clearer (Fig. 6). Other factors except electrical stimulation were controlled, such as cell density, culture medium (type or total volume), and even incubation or culture time.

The related sentences are at the line 63~92 in Page 4.

“~ In previous studies, electrical stimulation has been used to regulate cellular functions such as proliferation, migration, and differentiation of mesenchymal stem cells (MSCs) by modulating Ca^2+^ entry and activating calcineurin-NFAT signaling. ~”

*3) You mentioned the cytotoxicity of electrically stimulated KHYG-1 cells against breast cancer MCF-7 cells. Were other tumor cell lines tested, or was the experiment limited to MCF-7 cells? If other cell lines were used, were there any noticeable differences in cytotoxicity?*

**Response:** In this study, we analyzed the cytotoxicity of stimulated NK cells against two different breast cancer cell lines such as MCF-7 (actin response-negative) and MDA-MB-231 (actin response-positive) [5,6] . Electrical stimulated KHYG-1 cells showed 1.27-fold (0.5 V/cm) and 1.55-fold (1.0 V/cm) higher cytotoxicity against MCF-7, while the increase of cytotoxicity against MDA-MB-231 was not statistically significant. Even in the case of the breast cancer cells, it varies depending on the nature of the cells. For the different types of cancer cells, the degree of electrical stimulated KHYG-1 cells’ cytotoxic effects may vary depending on the characteristics of the target cancer cells. However, these results suggested that it would be worth to test the therapeutic potential against other cancer cells in further studies.

The related sentences are at the line 339~343 in Page 16.

“~ In the LDH cytotoxicity assays, changes in cytotoxicity against the MCF-7 cell line co-cultured with electrically stimulated NK cells appeared to be a cell line-specific response. The NK cell-mediated cytotoxicity of actin response-negative MCF-7 cells increased with electrically stimulated NK cells owing to their high susceptibility to NK cell-induced lysis but not in actin response-positive MDA-MB-231 cells.~”

*4) The study highlights the gene expression of granzyme B as a key factor in the increased cytotoxicity of electrically stimulated NK cells. Were there any other genes or proteins that showed significant changes in expression after electrical stimulation?*

**Response:** Our results point out that electrical stimulation has a significant impact on granzyme B gene expression, with 1.36-fold and 1.58-fold increases, and following consequent cytotoxicity. Expression for other cytotoxicity factors such as perforin, IFN-γ, TNF-α was analyzed, but was not significantly changed by electrical stimulation. In addition to the findings of this research, the expression of genes related to cell cytoskeleton remodeling (ACTB, CDC42) seemed to be upregulated after electrical stimulation (data not shown). This phenomenon could be studied further since it was reported that actin network and cytoskeletal structure remodeling could also play a critical role in immune synapse with tumors and release of cytolytic granules [6–8].

*5) How specific is the role of calcium influx in mediating the effects of electrical stimulation on NK cell function? Were there any other signaling pathways or factors that were explored in relation to NK cell activation and cytotoxicity?*

**Response:** Previous studies have reported that the role of calcium ions was important in immune cells related to its cytotoxic function and activity (especially to degranulation). As NFAT-mediated pathway is known as one of the major transcriptional regulators of activation in immune cells, there are other factors-mediated pathways such as Janus kinases- signal transducer and activator of transcription proteins (JAK-STAT) signaling and nuclear factor kappa-light-chain-enhancer of activated B cells (NF-κB) pathway [9,10]. NFAT-driven gene expression and immune cell immunity were highly dependent on sustained Ca^2+^ influx and calcineurin activity. Others, on the other hand, were less dependent on Ca^2+^ ions and calmodulin/calcineurin signaling pathway which was focused in this study with electrical stimulation [3,11–13]. In this respect, the results of this study also could support the association between intracellular Ca^2+^-related factors (especially in NFAT pathway) and cytolytic activity of NK cells with degranulation.

The related sentences are at the line 347~358 in Pages 16 ~ 17.

“~ In this process, the expression and degradation of secretory lysosomes are often accompanied by an increase in Ca^2+^ levels. Cytosolic Ca^2+^ concentration plays an important role in immune cells such as cytotoxic T cells and NK cells. Electrical stimulation alters Ca^2+^ influx and gene expression by mediating calcium signaling in various cell lines [25–27]. Based on these previous studies and experimental results, a further assay was conducted on Ca^2+^ to determine whether Ca^2+^ flux was related to the killing ability mediated by the degranulation of lytic granules in immune cells. The results of the Fluo-4 assay confirmed that stimulating cells in the electric field triggered an increase in intracellular Ca^2+^ (Fig 5A), possibly mediated by calcium channels [28], followed by activation of the calcineurin-NFAT signaling pathway (Fig 7), which was analyzed using RT-qPCR (Fig 5B). The results of this study suggested that a minor influx of Ca^2+^ (through electrical stimulation) had a positive effect on the anti-tumor cytotoxicity of NK cells by affecting transcriptional regulation through Ca^2+^ signaling (Fig 7). ~”

*6) The study focuses on breast cancer cells, but could the findings be applicable to other types of cancer as well? Is there any indication or speculation about the potential broader implications of this research in terms of cancer immunotherapy?*

**Response:** We evaluated the cytotoxicity of NK cells against two breast cell lines MCF-7 and more aggressive MDA-MB-231 cells, which showed the significantly different response to immunotherapy in previous studies [5,6]. As speculated with the results against MCF-7 cells in this study (about 53~90% cell cytotoxicity, Fig 3), it could be sufficiently applicable to leukemia and even other type of solid tumors.

The related sentences are at the line 225~228 in Page 11.

“~ The MDA-MB-231 cells with electrically stimulated KHYG-1 showed no significant increase in cytotoxicity (Fig 3A). However, the cytotoxicity of MCF-7 with electrically stimulated KHYG-1 cells increased from 53% to 74% (1.27-fold, 0.5 V/cm electrical stimulation) and from 58% to 90% (1.55-fold, 1.0 V/cm electrical stimulation), respectively. ~”

*7) Were there any potential limitations or challenges encountered during the study that may have influenced the results? Are there any aspects of the experimental design or methodology that should be taken into consideration when interpreting the findings?*

**Response:** It was not able to evaluate the real-time changes in intracellular calcium level while exposed to electrical stimulation. Although the result confirmed by the alternative method (analyzed at 45 min timepoint after stimulation) would not affect the results and interpretation of our findings, it was the challenges encountered during the study.

*8) Based on the findings of this study, what are the potential next steps or future research directions in exploring the use of electrical stimulation for enhancing NK cell function in immunotherapy? Are there any specific areas that warrant further investigation?*

**Response:** As the electrical stimulation have been a potential tool in tissue engineering, especially being efficient to stem cell proliferation and differentiation [2,14,15]. In immunotherapy in which area related to this study, further studies can be able to use this tool for cell culture inducing cell proliferation, activation, and cell phenotype by stimulating Ca^2+^-related transcriptional factor such as NFAT. Furthermore, it can also be applied to modulate immune system, enhancing immune cell proliferation, secretion of cytokines and degranulation [16,17].

The related sentences are at the line 59~72 in Pages 3~4.

***Reviewer #2’ s comments:***

*The authors investigated a new method for improving the functional potential of NK cells through electrical stimulation. This is a well written and structured research article. However, there are several issues with the results and discussion that need to be clarified/addressed. Below are more specific comments by section:*

*1)* ***Materials and methods. Western blotting:*** *The authors should clarify why 1.0 V/cm stimulation was chosen or be consistent and provide results for both 0.5 and 1.0 V/cm electrical stimulation.*

**Response:** We performed NFAT1 Western blot analysis using 1.0 V/cm stimulation samples, which are representative of stimulation conditions (possible maximum in this study), taking into account the results of cytotoxicity, granzyme B expression levels, and calcium signaling-related protein expression. Changes in granzyme B expression level and protein expression related to calcium signaling were observed in the range (0.5 ~ 1.0 V/cm) that did not affect cell viability. Similar response was described at #5) response.

We revised the corresponding sentences at the line 381~386 in Pages 18.

“ ~ NFAT1 dephosphorylation, mediated by the protein phosphatase calcineurin, was preceded by nuclear translocation, DNA-binding activity, and transcriptional regulation of gene expression (Fig 7). According to the western blotting results (Fig 6B), electrically stimulated (1.0 V/cm) KHYG-1 cells exhibited a 2.21-fold higher level of dephosphorylated NFAT1 than the control. This indicates that electrical stimulation induced the transcriptional activity of NFAT1 with Ca^2+^ influx and further transcriptional regulation of inducible target genes, such as granzyme B. ~”

*2)* ***Results. Effects of electrical stimulation on cell viability of NK cells:*** *The authors examine only viability, but not the ability to proliferate. For therapeutic use, NK cell proliferation is one of the most important factors, and adding this information to the article can be very valuable.*

**Response:** We evaluated the effect of electrical stimulation on cell viability with CCK-8 assay which could reflect mitochondrial activity, LDH release level, and BAX/BCL2 gene expression ratio at 24 and 48 hours after the stimulation. But as you suggested, we revised the Results section with the relevant information (lines 196-198), and the related data has been also included as S1 Fig. The CCK-8 assay results of NK cells at 24 h and 48 h after stimulation was over the 95 % of viability compared to control group (S1 fig). This result could suggest that there was no long-term negative effect of applied stimulation on cell viability of NK cells, and also no negative effects on the ability to proliferate.

*3)* ***Results. Effects of electrical stimulation on the cytotoxic activity of NK cells****: I suppose this is just a typo: “The MDA-MB-231 cells electrically stimulated with KHYG-1 showed no significant increase in cytotoxicity” and it should be “The MDA-MB-231 cells with electrically stimulated KHYG-1 showed no significant increase in cytotoxicity”. In this section, two co-culture times and two methods were mentioned: 24 hours and 6 hours. The authors should clarify this section to avoid confusion since it is not obvious what the reader should understand from Figure 3B and why 6 hours was used.*

**Response:** We apologized for some errors the reviewer indicated (lines 225-226). We revised the Results section and also double-checked similar mistakes throughout the manuscript. The cytotoxicity of NK cells against cancers was evaluated at 24 h of co-culture time. The live/dead cytotoxicity imaging was conducted at 6 h timepoint during co-culture because dead MCF-7 cells can be detached at the end of co-culture (24 h), which could make the results uncertain. As you suggested, we revised the Materials and Methods section, and the relevant information has been included.

We also revised the corresponding sentences at the line 120~122 in Pages 6.

“ ~ A live/dead cytotoxicity assay (L3224; Invitrogen) was used to measure the NK cell cytotoxicity at 6 h timepoint during co-culture, because most dead MCF-7 cells can be detached 24 hours after co-culture. ~”

*4)* ***Results. Fluo-4 calcium fluorescence assay****: The incubation time with Flow-4 AM was 45 min. This is confusing because the duration of the other experiments was 1 hour. If the authors can provide results with multiple time points such as 15, 30, 45, 60 minutes, it will increase the scientific value of this work, otherwise the authors should be consistent and use the same time for all experiments.*

**Response:** According to the manufacturer’s protocol, the incubation time for Fluo-4 AM dye staining at 37 °C was up to 45 minutes. Following the recommended instruction, we carried out with maximum staining time while carrying electrical stimulation. The calcium assay for multiple timepoint could not be conducted simultaneously because of the condition with stimulation system. Therefore, calcium fluorescence assay was conducted only with 45 minutes also with consideration of plate-to-plate variation of each timepoint. We added additional explanations in Materials and Methods section of current manuscript.

We revised the corresponding sentences at the line 157~159 in Pages 8.

“~ KHYG-1 cells were incubated with Fluo-4 AM dye for 45 min at 37 °C while being exposed to each voltage range of electrical stimulation, which was performed during the maximum time recommended in the manufacturer’s protocol for staining dye.~”

*5)* ***Discussion.*** *The difference in results between the 0.5 and 1.0 V/cm groups needs to be clarified. The authors should discuss why Figure 5a shows that Ca^2+^ levels are higher for the 0.5 V/cm group and cytotoxicity is higher for the 1 V/cm group.*

**Response:** The first point the reviewer mentioned was about the differences in results between 0.5 V/cm and 1.0 V/cm stimulated group. In our study, the increase in intracellular calcium level induced by stimulation seemed not be always proportional to the strength of applied voltage, as other previous studies had suggested [18]. The second point the reviewer indicated was about the cytotoxicity of stimulated NK cells, which showed higher results in 1.0 V/cm group. In this study, there was optimum range of Ca^2+^ level, and the effects mediated by 1.0 V/cm condition could be efficient for inducing granulation of NK cells at this range. The range (0.5 ~ 1.0 V/cm) did not affect cell viability.

*6)* ***Discussion.*** *For the concentration of granzyme B intracellular concentration is higher for 1 V/cm but for culture media the situation is reverse at 4h. The concentration of granzyme B after 8 hours is the same for all three groups. This fact may lead to the conclusion that electrical stimulation has a short-term effect and cannot be considered as a potential treatment, and all differences can be explained by the effect within the first 4 hours and the fact that MCF-7 cell line is susceptible for the KHYG-1 cells that is highly cytotoxic cell line.*

**Response:** The expression of granzyme B gene increased 1.36-fold and 1.58-fold, which also led to actual increase in intracellular protein expression detected by ELISA assay (Fig 4B). The expressed granzyme B protein was pre-stored in NK cells and were not secreted much during 4 h or 8 h incubation without co-culture of tumor cells. NK cells display stronger degranulation responses after forming in contact with tumor cells [8,19,20]. No difference between control and stimulated NK cells after 8 h was due to absence of tumor cells in the assay, although stimulated NK cells contained 1.26-fold higher pre-stored granzyme B protein as intracellular granzyme B level. However, with the data for 24 h of co-culture (Fig 3), it seemed significant that the stimulation could enhance the cytolytic activity of NK cells with the expected long-term effects.

The related sentences are at the line 248~251 in Pages 12.

“~ ELISA was used to analyze granzyme B protein expression (Fig 4B). Upregulated Granzyme B expression leads to changes in protein expression. The intracellular levels of granzyme B in electrically stimulated KHYG-1 cells were high at 0.5 V/cm and significantly higher at 1.0 V/cm (1.26-fold change). Granzyme B secreted into the culture medium was sampled at 4 and 8 h after electrical stimulation.~”

*7)* ***Discussion.*** *Another question is why 1h co-incubation was selected, what would happened with different incubation time?*

**Response:** We analyzed the cell viability after being exposed to electrical stimulation and optimized the stimulation conditions in a relatively low range (0.5-1.0 V/cm) for 1 h so as not to affected by the generation of heat or bubbles. However, due to the drawbacks of the direct coupling method of electrical stimulation, it should be verified with long-term duration of stimulation, which could result in elevated temperature, pH changes, and the generation of by-products such as reactive oxygen species (ROS) (ex. H_2_O_2_) [21].

**References**

1.	Thrivikraman G, Boda SK, Basu B. Unraveling the mechanistic effects of electric field stimulation towards directing stem cell fate and function: A tissue engineering perspective. Biomaterials. 2018;150: 60–86. doi:10.1016/j.biomaterials.2017.10.003

2.	Srirussamee K, Xue R, Mobini S, Cassidy NJ, Cartmell SH. Changes in the extracellular microenvironment and osteogenic responses of mesenchymal stem/stromal cells induced by in vitro direct electrical stimulation. J Tissue Eng. 2021;12: 204173142097414. doi:10.1177/2041731420974147

3.	Zhao H, Steiger A, Nohner M, Ye H. Specific Intensity Direct Current (DC) Electric Field Improves Neural Stem Cell Migration and Enhances Differentiation towards βIII-Tubulin+ Neurons. Hu W, editor. PLoS One. 2015;10: e0129625. doi:10.1371/journal.pone.0129625

4.	Kumar A, Nune KC, Misra RDK. Electric field-mediated growth of osteoblasts-the significant impact of dynamic flow of medium. Biomater Sci. 2016;4: 136–144. doi:10.1039/c5bm00350d

5.	Al Absi A, Wurzer H, Guerin C, Hoffmann C, Moreau F, Mao X, et al. Actin Cytoskeleton Remodeling Drives Breast Cancer Cell Escape from Natural Killer-Mediated Cytotoxicity. Cancer Res. 2018;78: 5631–5643. doi:10.1158/0008-5472.CAN-18-0441

6.	Lee M, Kim D, Kwon S. Hypergravity-induced changes in actin response of breast cancer cells to natural killer cells. Sci Rep. 2021;11: 7267. doi:10.1038/s41598-021-86799-7

7.	Burkhardt JK. Cytoskeletal function in the immune system. Immunol Rev. 2013;256: 5–9. doi:10.1111/imr.12121

8.	Rak GD, Mace EM, Banerjee PP, Svitkina T, Orange JS. Natural killer cell lytic granule secretion occurs through a pervasive actin network at the immune synapse. Marrack P, editor. PLoS Biol. 2011;9: e1001151. doi:10.1371/journal.pbio.1001151

9.	Schindler C, Darnell JE. Transcriptional responses to polypeptide ligands: The JAK-STAT pathway. Annu Rev Biochem. 1995;64: 621–651. doi:10.1146/annurev.bi.64.070195.003201

10.	Crabtree GR, Clipstone NA. Signal transmission between the plasma membrane and nucleus of T lymphocytes. Annu Rev Biochem. 1994;63: 1045–83. doi:10.1146/annurev.bi.63.070194.005145

11.	Kaschek L, Zöphel S, Knörck A, Hoth M. A calcium optimum for cytotoxic T lymphocyte and natural killer cell cytotoxicity. Semin Cell Dev Biol. 2021;115: 10–18. doi:10.1016/j.semcdb.2020.12.002

12.	Pores-Fernando AT, Zweifach A. Calcium influx and signaling in cytotoxic T-lymphocyte lytic granule exocytosis. Immunol Rev. 2009;231: 160–173. doi:10.1111/j.1600-065X.2009.00809.x

13.	Huseby ES, Teixeiro E. The perception and response of T cells to a changing environment are based on the law of initial value. Sci Signal. 2022;15: eabj9842. doi:10.1126/scisignal.abj9842

14.	Mobini S, Leppik L, Parameswaran VT, Barker JH. In vitro effect of direct current electrical stimulation on rat mesenchymal stem cells. PeerJ. 2017;2017: e2821. doi:10.7717/peerj.2821

15.	Amini AR, Laurencin CT, Nukavarapu SP. Bone tissue engineering: Recent advances and challenges. Crit Rev Biomed Eng. 2012;40: 363–408. doi:10.1615/CritRevBiomedEng.v40.i5.10

16.	Das R, Langou S, Le TT, Prasad P, Lin F, Nguyen TD. Electrical Stimulation for Immune Modulation in Cancer Treatments. Front Bioeng Biotechnol. 2022;9: 1–18. doi:10.3389/fbioe.2021.795300

17.	Arnold CE, Rajnicek AM, Hoare JI, Pokharel SM, Mccaig CD, Barker RN, et al. Physiological strength electric fields modulate human T cell activation and polarisation. Sci Rep. 2019;9: 17604. doi:10.1038/s41598-019-53898-5

18.	Khatib L, Golan DE, Cho M. Physiologic electrical stimulation provokes intracellular calcium increase mediated by phospholipase C activation in human osteoblasts. FASEB J. 2004;18: 1903–1905. doi:10.1096/fj.04-1814fje

19.	Brown ACN, Oddos S, Dobbie IM, Alakoskela JM, Parton RM, Eissmann P, et al. Remodelling of cortical actin where lytic granules dock at Natural Killer cell immune synapses revealed by super-resolution microscopy. Marrack P, editor. PLoS Biol. 2011;9: e1001152. doi:10.1371/journal.pbio.1001152

20.	Goodridge JP, Jacobs B, Saetersmoen ML, Clement D, Hammer Q, Clancy T, et al. Remodeling of secretory lysosomes during education tunes functional potential in NK cells. Nat Commun. 2019;10: 514. doi:10.1038/s41467-019-08384-x

21.	Chen C, Bai X, Ding Y, Lee I-S. Electrical stimulation as a novel tool for regulating cell behavior in tissue engineering. Biomater Res. 2019;23: 25. doi:10.1186/s40824-019-0176-8
